# Characterization of Vestibular Phenotypes in Patients with Genetic Hearing Loss

**DOI:** 10.3390/jcm13072001

**Published:** 2024-03-29

**Authors:** Ji Hyuk Han, Seong Hoon Bae, Sun Young Joo, Jung Ah Kim, Se Jin Kim, Seung Hyun Jang, Dongju Won, Heon Yung Gee, Jae Young Choi, Jinsei Jung, Sung Huhn Kim

**Affiliations:** 1 Department of Otorhinolaryngology, Yonsei University College of Medicine, Seoul 03722, Republic of Korea; jihyukhan.md@gmail.com (J.H.H.); jychoi@yuhs.ac (J.Y.C.); 2 Department of Pharmacology, Graduate School of Medical Science, Brain Korea 21 Project, Yonsei University College of Medicine, Seoul 03722, Republic of Korea; kin2844@yuhs.ac (S.Y.J.); wjddk97@yuhs.ac (J.A.K.); tpwls777@yuhs.ac (S.J.K.); tkdnsel93@yuhs.ac (S.H.J.); hygee@yuhs.ac (H.Y.G.); 3 Department of Laboratory Medicine, Yonsei University College of Medicine, Seoul 03722, Republic of Korea; wdjbabo@yuhs.ac

**Keywords:** genetic variation, inheritance pattern, vestibular function test, vertigo

## Abstract

**Background:** The vestibular phenotypes of patients with genetic hearing loss are poorly understood. **Methods:** we performed genetic testing including exome sequencing and vestibular function tests to investigate vestibular phenotypes and functions in patients with genetic hearing loss. **Results:** Among 627 patients, 143 (22.8%) had vestibular symptoms. Genetic variations were confirmed in 45 (31.5%) of the 143 patients. Nineteen deafness genes were linked with vestibular symptoms; the most frequent genes in autosomal dominant and recessive individuals were *COCH* and *SLC26A4*, respectively. Vestibular symptoms were mostly of the vertigo type, recurrent, and persisted for hours in the genetically confirmed and unconfirmed groups. Decreased vestibular function in the caloric test, video head impulse test, cervical vestibular-evoked myogenic potential, and ocular vestibular-evoked myogenic potential was observed in 42.0%, 16.3%, 57.8%, and 85.0% of the patients, respectively. The caloric test revealed a significantly higher incidence of abnormal results in autosomal recessive individuals than in autosomal dominant individuals (*p* = 0.011). The genes, including *SLC26A4*, *COCH*, *KCNQ4*, *MYH9*, *NLRP3*, *EYA4*, *MYO7A*, *MYO15A*, and *MYH9*, were heterogeneously associated with abnormalities in the vestibular function test. **Conclusions:** In conclusion, diverse vestibular symptoms are commonly concomitant with genetic hearing loss and are easily overlooked.

## 1. Introduction

The rate of permanent hearing loss in neonates varies from 0.1% to 0.2% [1,2]. In developed countries, 50–60% of childhood hearing loss has genetic causes [3,4]. Most cases of genetic hearing loss have a non-syndromic and autosomal recessive (AR) inheritance mode [5]. Recent studies on genetic hearing loss have greatly increased the understanding of the auditory function and pathophysiological processes of hearing loss. To date, 124 non-syndromic hearing loss genes and 223 deafness-associated genes and their variants have been identified (https://hereditaryhearingloss.org and by https://deafnessvariationdatabase.org, accessed on 1 December 2023) [6,7,8].

Compared with hereditary hearing loss, relatively little is known about the genetic causes of vestibular disorders. The vestibular organ has cellular structures that share histological and functional homologies with those in the ear cochlea, thereby making possible the speculation that variations causing hearing loss may also induce vestibular phenotypes, such as dizziness, vertigo, and disequilibrium [9]. Although symptoms can vary, the vestibular phenotypes in patients with genetic hearing loss are manageable because central compensation mechanisms typically alleviate them, and symptoms are unlikely to be severe unless a patient has a complete bilateral vestibular loss [10]. However, even with incomplete loss of vestibular function, patients can be uncomfortable and sometimes disabled during fast and abrupt movements accompanying high acceleration. Moreover, recurrent vertigo spells critically impacting daily life have been reported in patients with the *SLC26A4* and *COCH* variations [11,12,13,14].

Fewer than 20 genes responsible for vestibular disorders and hearing loss have been identified. However, the vestibular phenotypes and functions in patients with variations in these genes are poorly characterized [9,11,12,13,14,15,16,17,18,19,20,21,22,23,24,25,26,27,28,29,30,31]. Additionally, more genes and variations should be expected in cases of genetic hearing loss with vestibular phenotypes because of the involvement of multiple cochlear and vestibular epithelial and neuronal cells. In this study, we investigate the prevalence and clinical characteristics of vestibular symptoms in a cohort of patients with hereditary hearing loss. We also evaluate the genetic factors associated with vestibular symptoms.

## 2. Materials and Methods

### 2.1. Study Design and Participants

This cross-sectional study involved recruiting patients registered in the hereditary hearing loss cohorts of our institution, named the Yonsei University Hearing Loss (YUHL) and Yonsei University Enlarged Vestibular Aqueduct (YUEVA) cohorts, including patients with enlarged vestibular aqueducts observed through temporal bone computed tomography scans. Patients were included in these cohorts if they had bilateral hearing loss with a definite family history or had undergone genetic testing. Patients were excluded based on the following criteria: (1) age < 6 years, (2) positional vertigo and high suspicion of benign paroxysmal positional vertigo, and (3) vestibular symptoms of central origin. This study was approved by the Institutional Review Board of our hospital (approval no. 4-2015-0659). Written informed consent was obtained from all participants.

### 2.2. Acquisition of Information about Vestibular Phenotypes

In routine clinical settings, patients who reported dizziness were interviewed via our survey form to investigate the characteristics and types, onset, duration, recurrence, and frequency of their dizziness; associated symptoms such as headaches, nausea, and vomiting; aggravating or relieving factors; associated ear symptoms such as tinnitus or hearing loss; and other risk factors. Dizziness was categorized as vertigo, unsteadiness, or other non-vertiginous dizziness. Vertigo refers to the sensation of self-motion or distorted self-motion during an otherwise normal head movement, based on the classification of vestibular symptoms provided by the Barany Society [32]. We categorized the duration as follows: persisting dizziness (particularly when moving) (i.e., continuous); >1 day (days); >1 h and <1 day (hours); several minutes and <1 h (minutes); and <1 min (seconds). Telephone interviews were conducted only for patients whose information was inadequately acquired during the initial interview.

### 2.3. Evaluation of Hearing and Vestibular Function

All patients underwent pure-tone audiometry during screening. The hearing threshold was calculated as the threshold average at 500, 1000, 2000, and 4000 Hz. Vestibular function tests, including the bithermal caloric test (Micromedical Technology Inc., Calabasas, CA, USA), video head impulse test (vHIT) (ICS Impulse, Natus, Pleasanton, CA, USA), and cervical/ocular vestibular-evoked myogenic potential (c/oVEMP) test (Bio-logic Navigator Pro; Natus, Pleasanton, CA, USA), were performed only in patients who consented to and tolerated the tests. We regarded vestibular function as “abnormal” if the canal paresis value in the caloric test was >25%, if catch-up saccade occurred with decreased gain in the vHIT, or if sound-evoked electromyographic waves were absent in the c/oVEMP tests.

### 2.4. Genetic Analyses

All individuals enrolled in the YUHL study underwent genetic testing. Patients underwent two-track genetic testing of panel sequencing or whole-exome sequencing (WES), based on their willingness, given that payment was covered by the national government insurance system [14,33,34,35]. For the panel next-generation sequencing, a panel of 207 deafness genes was customized with validated evidence based on an extensive literature review, the Hereditary Hearing Loss Homepage database (https://hereditaryhearingloss.org/, accessed on 13 October 2023), the Deafness Variation Database (http://deafnessvariationdatabase.org/, accessed on 13 October 2023), and the Online Mendelian Inheritance in Man database [35] (Supplementary Table S1). For WES, pre-capture libraries were constructed using the Agilent SureSelect V5 enrichment capture kit (Agilent Technologies, Santa Clara, CA, USA) based on the manufacturer’s sample preparation protocol. Pooled libraries were sequenced using the MiSeq sequencer (Illumina, San Diego, CA, USA) and the MiSeq Reagent Kit v2 (300 cycles). The segregation of candidate genes, identified using next-generation sequencing or WES, was examined through Sanger sequencing of family members or siblings with or without hearing loss. Variants with a minimum coverage of 20, a minimum count of 5, and a minimum frequency of 20% were detected using the “Basic Variant Caller” function in CLC. Variants with minor allele frequencies of >0.5% and >0.05% for recessive and dominant hearing loss genes, respectively, in the dbSNP database (v153, https://www.ncbi.nlm.nih.gov/snp/ accessed on 13 October 2023) and gnomAD (v4.0.0, https://gnomad.broadinstitute.org/ accessed on 13 October 2023) were excluded. Chromosomal copy number variations were detected using the EXCAVATOR (version 2.2) and ExomeDepth tools (version 1.1.10) with their default settings. Genetic diagnoses were determined by a board of otolaryngologists, geneticists, laboratory personnel, and bioinformaticians, per the hearing-loss-specified American College of Medical Genetics and Genomics guidelines in the Deafness Variation Database [36]. Targeted Sanger sequencing for whole exons and exon–intron junctions of *SLC26A4* was conducted for the YUEVA cohort.

### 2.5. Statistical Analyses

Statistical data analysis was conducted using SPSS software (version 25; IBM, Armonk, NY, USA). A *p*-value < 0.05 was considered statistically significant. In this study, the age of onset was the only continuous variable that was analyzed. There were no outliers. Continuous data are presented as the mean with standard deviation and were analyzed using the *t*-test. We used the Shapiro–Wilk test and Q–Q plots to decide between normal and non-normal data, and the Levene test to check the homogeneity of the variance. When the assumptions were violated, we used the Mann–Whitney U test for comparison. One comparison (confirmed vs. unconfirmed in the AR group) did not exhibit a normal distribution and was analyzed with the Mann–Whitney U test. Discrete data are presented as the count, number, and proportion and were analyzed using the chi-square or Fisher’s exact tests. Chi-square or Fisher’s exact tests were used to analyze symptom types, symptom recurrence, the duration of vestibular symptoms, and the results of the vestibular function tests, including the caloric test, vHIT, cVEMP test, and oVEMP test. Post hoc multiple comparison analysis was performed with Bonferroni correction. Furthermore, we performed logistic regression on the caloric test results, which revealed significant differences in the descriptive analysis.

## 3. Results

### 3.1. Clinical Characteristics and Vestibular Phenotypes of the Total Study Population

**Figure 1:**
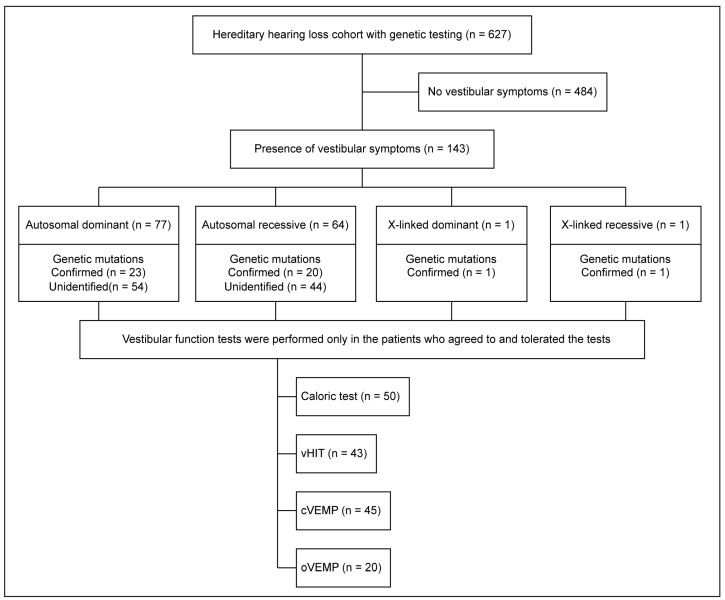
Flow chart for patient selection, the number of patients with confirmed genetic mutations, and the number of patients who underwent each vestibular function test. cVEMP, cervical vestibular-evoked myogenic potential; oVEMP, ocular vestibular-evoked myogenic potential; vHIT, video head impulse test.

Of the 627 patients enrolled in the YUHL cohort, 143 (22.8%) patients exhibited vestibular symptoms (Figure 1). The mean age of onset in patients with vestibular phenotypes was 37.9 ± 18.6 years (Table 1), and the data revealed a female preponderance (male–female = 55:88). Most patients (n = 65, 62.5%) experienced vertigo-type dizziness. Most vestibular symptoms recurred (n = 87, 90.6%) and persisted for several hours (n = 31, 33.7%). The numbers of patients who underwent the caloric test, vHIT, cVEMP test, and oVEMP test were 50, 43, 45, and 20, respectively. Each test showed decreased vestibular function in 21 (42.0%), 7 (16.3%), 26 (57.8%), and 17 (85.0%) patients, respectively. The most common inheritance pattern was autosomal dominant (AD; n = 77, 53.8%), followed by autosomal recessive (AR; n = 64, 44.8%), X-linked dominant (XD; n = 1, 0.7%), and X-linked recessive (XR; n = 1, 0.7%). Only one patient had XD and XR inheritance; thus, the vestibular phenotypes of AD and AR inheritance patients were further analyzed.

### 3.2. Vestibular Phenotypes, Based on Inheritance Pattern, among Patients with Confirmed or Unconfirmed Genetic Variations

Genetic variations were confirmed in 45 (31.5%) of the 143 patients. Vestibular symptoms and the vestibular function test findings were compared between patients with AD and AR inheritance (Table 1). The age of onset was 41.6 ± 17.1 and 33.1 ± 19.5 in the AD and AR groups, respectively (*p* = 0.027, Cohen’s d: 0.46, confidence interval: 0.05–0.88). The characteristics of the vestibular symptoms were mostly of the recurrent vertigo type in each subgroup, which insignificantly differed between the genetically confirmed and unconfirmed cases in patients with AD and AR inheritance and between the AD and AR groups. In the AR group, symptoms lasting for days were more frequently observed in genetically confirmed cases than in unconfirmed cases (*p* = 0.043, Cramer’s V: 0.30, confidence interval: 0.00–0.63). However, multiple comparisons with Bonferroni correction did not reveal any significant differences among the groups. The frequency of abnormal caloric test results was significantly higher in patients with AR inheritance than in those with AD inheritance (*p* = 0.011, Cramer’s V: 0.38, confidence interval: 0.00–0.67). However, the frequencies of abnormal results of the vHIT, cVEMP test, and oVEMP test insignificantly differed between the AD and AR groups (vHIT, *p* = 0.420; cVEMP, *p* = 1.000; and oVEMP, *p* = 0.270). The abnormal results of the vestibular function tests between genetically confirmed and unconfirmed cases differed insignificantly in the AD and AR subgroups (AD: caloric test, *p* = 0.348; vHIT, *p* = 0.297; cVEMP, *p* = 0.089; oVEMP, *p* = 1.000; AR: caloric test, *p* = 0.412; vHIT, *p* = 1.000; cVEMP, *p* = 0.187; oVEMP, *p* = 1.000).

### 3.3. Logistic Regression for Caloric Test Results

We further conducted logistic regression on the caloric test results because only this test showed differences in the inheritance patterns. After excluding participants with missing data, the number of analyzed patients was 37. Onset age, sex, and genetic diagnosis of hereditary hearing loss were insignificant factors (onset age, *p* = 0.299; sex, *p* = 0.132; and genetic diagnosis, *p* = 0.159) (Table 2). However, the AR inheritance pattern was a significant factor for caloric weakness (β = 3.127, SE = 1.107, *p* = 0.005).

### 3.4. Genetic Variations Associated with Vestibular Phenotypes

Table 3 presents the vestibular phenotypes based on genetic variation. Among all study participants, 19 genes harbored variations in 45 patients. Vestibular symptoms differed uncharacteristically, depending on the individual causative genes. Association studies between genetic variations and vestibular symptoms revealed the following crucial findings: (1) caloric weakness was primarily linked to *COCH*, *EYA4*, *MYH9*, and *NLRP3* in AD individuals and *MYO15A* and *SLC26A4* in AR individuals; (2) the major genes associated with abnormal vHIT results were *COCH*, *EYA4*, and *KCNQ4* in AD individuals; (3) the major genes associated with abnormal cVEMP results were *COCH*, *EYA4*, *KCNQ4*, *MYH9*, and *MYO7A* in AD individuals and *CDH23*, *SLC26A4*, and *MYO15A* in AR individuals; (4) for abnormal oVEMP results, the linked genes were *COCH*, *MYH9*, and *MYO7A* in AD individuals and *SLC26A4* in AR individuals; (5) total bilateral cVEMP and oVEMP abnormalities were found in *COCH*, *MYH9*, and *MYO7A* in AD individuals; and (6) total bilateral loss in all tests was found only in patients with *COCH* variations. When the abnormal results of the vestibular function tests were categorized based on the laterality of the vestibulopathy (Table 4), the caloric test tended to be unilaterally abnormal, whereas the cVEMP and oVEMP tests tended to be bilateral (*p* < 0.001, Cramer’s V: 0.52, confidence interval: 0.21–0.75). After the post hoc multiple comparison with Bonferroni correction, significant differences were observed in the comparisons between the caloric test and cVEMP test (*p* < 0.001), as well as between the caloric test and oVEMP test (*p* = 0.002). This finding indicated that canalopathy, called “canal paresis” in the caloric test, occurs unilaterally, whereas otolithic pathology occurs bilaterally. The identified variants and genotypes are listed in Supplementary Table S2.

## 4. Discussion

To the best of our knowledge, this is the first comprehensive study to investigate the vestibular phenotypes of patients with hereditary hearing loss using data from a large patient population. Furthermore, we investigated the clinical characteristics of the vestibular phenotypes of the identified genes. Although vestibular phenotypes with hereditary hearing loss have been reported previously [9,11,12,13,14,15,16,17,18,19,20,21,22,23,24,25,26,27,28,29,30,31], most of these studies enrolled few patients or described vestibular symptoms/results of vestibular function tests that focused on specific genes causing hearing loss.

This study has the following salient findings. First, more than 20% of patients with hereditary hearing loss had varying degrees of vestibular symptoms, the most common of which were recurrent vertigo and vestibular symptoms persisting for hours. Vestibular symptoms did not differ between inheritance patterns or mutated genes. Second, patients who underwent clinical vestibular function tests exhibited varying degrees of vestibular dysfunction. Moreover, abnormal caloric test results were significantly associated with AR inheritance patterns, indicating that unilateral canalopathy was more prevalent among AR patients. Third, several genetic variations causing vestibular symptoms that have remained unreported in the literature were identified in this study.

Weiner–Vacher et al. [37] recently reported that >50% of children with hearing loss have a vestibular impairment, and 20% have bilateral vestibular loss. In contrast, our study showed that >20% of patients with hereditary hearing loss experienced dizziness and vertigo. The parameters used for the vestibular phenotypes and patient enrollment criteria in the work by Weiner–Vacher et al. differed from those in our study; therefore, the results of the two studies cannot be directly compared. In the Weiner–Vacher et al. study, the enrolled patients were only children with congenital hearing loss, which did not necessarily have hereditary etiologies (e.g., cytomegalovirus infection and inner-ear malformation), whereas our study enrolled patients of all ages who were eligible for vestibular function testing and only hereditary cases were considered. Therefore, a higher proportion of vestibular phenotypes in the Weiner–Vacher et al. study may have been attributed to the inclusion of young children with a broader spectrum of congenital hearing loss, in addition to that of hereditary origin [37].

Our study also revealed a relatively high proportion of vestibular phenotypes in patients with genetic hearing loss. This finding may confer a common genetic predisposition to vestibular and auditory functions. Although the mechanical stimuli differ between the two organs, functionally and differentially expressed genes could be shared between the two organs, and the homology of the cochlear and vestibular apparatuses may result in the high comorbidity of hearing loss and vestibular dysfunction. Recently, it was revealed that familial Meniere’s disease affects both cochlear and vestibular organs through the enriched genetic burden of rare variants in *OTOG* and *GJD3* [38,39]. These findings indicate that the characteristic of vestibular impairment is associated with that of cochlear impairment via genetic predisposition.

Vertigo characteristics and the degree of vestibular dysfunction can describe vestibular phenotypes in patients with genetic hearing loss. Although long-term experiences of recurrence and progression in vestibular attacks can induce vestibular dysfunction, they are not always correlated, as observed in patients with Meniere’s disease [40]. Therefore, symptoms and vestibular function test results should be simultaneously investigated to identify vestibular phenotypes. The vestibular function test results here revealed that unilateral canalopathy was more common than bilateral canalopathy in the caloric test, whereas bilateral otolith dysfunction was more prevalent than unilateral dysfunction in the VEMP tests. These findings suggest that bilateral canalopathy, a devastating condition causing severe handicaps relating to balance and gait, is rare, even in genetic forms of vestibular dysfunction. The caloric responses were similarly decreased unilaterally in patients with the AR inheritance pattern. This finding may be attributable to the high incidence of unilateral deformity in the semicircular canal function during development, a more vulnerable period in the AR pattern [41].

In our study, 19 mutated deafness genes associated with vestibular phenotypes were identified, and AD was the most common inheritance pattern. Variations in *COCH*, *MYO7A*, *TECTA*, *WFS1*, and *POU4F3* with AD inheritance patterns and *CDH23*, *GJB2*, and *SLC26A4* with AR inheritance patterns are associated with vestibular phenotypes [6,9,13,14,15,28,29,30,31,42,43]. However, other genes such as *DIAPH1*, *PMXL2*, *EYA4*, *GRHL2*, *KCNQ4*, *MYH9,* and *NLRP3* with AD inheritance patterns and *MPZL2*, *MYO15A*, and *WFS1* with AR inheritance patterns have not been reported regarding their association with vestibular phenotypes. Furthermore, the X-linked inherited mutated genes *COL4A6* and *PRPS1* have not been reported to be associated with vestibular phenotypes. The RNA expression of *DIAPH1*, *DMXL2*, *EYA4*, *GRHL2*, *MYH9*, *NLRP3*, *MPZL2*, *COL4A6*, or *PRPS1* in the vestibule was notably identified in murine vestibular organs (Supplementary Table S3). Although it is unclear which types of cells express such genes in the vestibular system, we reason that genetic variants of those genes may cause progressive degeneration of the sensory and supporting cells in semicircular canals and otolith organs, resulting in abnormal findings in vestibular function tests. However, the protein expression of these genes in the vestibular organ remains unvalidated, and whether such variations cause gain-of-function or loss-of-function contributions to vestibular dysfunction remains unclear. Given that these genes are involved in maintaining cellular structures, immune reactions, neurotransmission, and development in the cochlea, they may also contribute to the development and maintenance of vestibular organs and their functions. Studies on the distribution of proteins encoded by these genes and their roles should be conducted in human genetic hearing loss cohorts and animal models.

This study had some limitations. First, we reviewed medical records retrospectively. There were 36 patients who were interviewed via telephone due to inadequate information about their vestibular phenotypes. However, even with the telephone interviews, there were missing data, as shown in Table 1. Second, this study included relatively few patients who underwent vestibular function tests because only patients who agreed to undergo the tests were enrolled. Owing to these limitations, the frequency of vestibular symptoms and vestibular impairment may have been underestimated. Third, there could be confounding factors such as socioeconomic status or psychological factors, which were not assessed in this study. Fourth, the results of this study have limited generalizability because it was conducted in a single tertiary center. In different geographic or demographic groups, the results may not be representative.

In conclusion, we identified common vestibular symptoms in patients with hereditary hearing loss and found that vestibular dysfunction was commonly associated with genetic hearing loss. Therefore, vestibular evaluations should be performed more enthusiastically to ensure vestibular rehabilitation and therapy in patients with inherited hearing loss.

## Figures and Tables

**Table 1:** Demographic data and vestibular phenotypes of the enrolled patients.

	Total (n = 143)	AD				AR				*p*-Value (AD vs. AR)
		Total (n = 77)	Confirmed (n = 23)	Unconfirmed (n = 54)	*p*-Value	Total (n = 64)	Confirmed (n = 20)	Unconfirmed (n = 44)	*p*-Value	
Age of onset	37.9 ± 18.6	41.6 ± 17.1	38.1 ± 14.2	43.0 ± 18.2	0.344	33.1 ± 19.5	25.6 ± 21.1	34.9 ± 19.0	0.168	* 0.027
Sex					1.000				1.000	0.229
Male	55 (38.5%)	25 (32.5%)	7 (30.4%)	18 (33.3%)		28 (43.8%)	9 (45.0%)	19 (43.2%)		
Female	88 (61.5%)	52 (67.5%)	16 (69.6%)	36 (66.7%)		36 (56.2%)	11 (55.0%)	25 (56.8%)		
Type of symptoms					0.222				1.000	0.055
Vertigo	65 (62.5%)	35 (59.3%)	8 (47.1%)	27 (64.3%)		30 (66.7%)	5 (71.4%)	25 (65.8%)		
Non-vertiginous dizziness	32 (30.8%)	17 (28.8%)	5 (29.4%)	12 (28.6%)		15 (33.3%)	2 (28.6%)	13 (34.2%)		
Unsteadiness	7 (6.7%)	7 (11.9%)	4 (23.5%)	3 (7.1%)		0 (0.0%)	0 (0.0%)	0 (0.0%)		
Recurrence					1.000				1.000	0.727
Recurrent	87 (90.6%)	48 (88.9%)	13 (86.7%)	35 (89.7%)		39 (92.9%)	9 (90.0%)	30 (93.8%)		
Single episode	9 (9.4%)	6 (11.1%)	2 (13.3%)	4 (10.3%)		3 (7.1%)	1 (10.0%)	2 (6.2%)		
Duration					0.079				* 0.043	0.702
Continuous	3 (3.3%)	3 (5.5%)	3 (17.6%)	0 (0.0%)		0 (0.0%)	0 (0.0%)	0 (0.0%)		
Days	22 (23.9%)	12 (21.8%)	3 (17.6%)	9 (23.7%)		10 (27.0%)	4 (57.1%)	6 (20.0%)		
Hours	31 (33.7%)	18 (32.7%)	7 (41.2%)	11 (28.9%)		13 (35.1%)	0 (0.0%)	13 (43.3%)		
Minutes	26 (28.3%)	15 (27.3%)	3 (17.6%)	12 (31.6%)		11 (29.7%)	2 (28.6%)	9 (30.0%)		
Seconds	10 (10.9%)	7 (12.7%)	1 (5.9%)	6 (15.8%)		3 (8.1%)	1 (14.3%)	2 (6.7%)		
Caloric test					0.348				0.412	* 0.011
Normal	29 (58.0%)	20 (76.9%)	7 (63.6%)	13 (86.7%)		9 (37.5%)	4 (50.0%)	5 (31.2%)		
Abnormal	21 (42.0%)	6 (23.1%)	4 (36.4%)	2 (13.3%)		15 (62.5%)	4 (50.0%)	11 (68.8%)		
vHIT					0.297				1.000	0.420
Normal	36 (83.7%)	18 (78.3%)	5 (62.5%)	13 (86.7%)		18 (90.0%)	6 (100.0%)	12 (85.7%)		
Abnormal	7 (16.3%)	5 (21.7%)	3 (37.5%)	2 (13.3%)		2 (10.0%)	0 (0.0%)	2 (14.3%)		
cVEMP test					0.089				0.187	1.000
Normal	19 (42.2%)	10 (43.5%)	1 (14.3%)	9 (56.2%)		9 (40.9%)	5 (62.5%)	4 (28.6%)		
Abnormal	26 (57.8%)	13 (56.5%)	6 (85.7%)	7 (43.8%)		13 (59.1%)	3 (37.5%)	10 (71.4%)		
oVEMP test					1.000				1.000	0.270
Normal	3 (15.0%)	1 (7.7%)	0 (0.0%)	1 (10.0%)		2 (28.6%)	1 (50.0%)	1 (20.0%)		
Abnormal	17 (85.0%)	12 (92.3%)	3 (100.0%)	9 (90.0%)		5 (71.4%)	1 (50.0%)	4 (80.0%)		

AD, autosomal dominant; AR, autosomal recessive; cVEMP, cervical vestibular-evoked myogenic potential; N/A, not available; oVEMP, ocular vestibular-evoked myogenic potential; vHIT, video head impulse test. * *p*-value less than 0.05.

**Table 2:** Logistic regression for the results of the caloric tests.

	Estimate	SE	*p*-Value	Odds Ratio	Confidence Interval
Age of onset	0.030	0.029	0.299	1.03	0.97–1.09
Sex					
Male	Reference				
Female	1.500	0.995	0.132	4.48	0.64–31.52
Genetic variation					
Unidentified	Reference				
Confirmed	1.418	1.007	0.159	4.13	0.57–29.72
Inheritance pattern					
AD	Reference				
AR	3.127	1.107	0.005	22.80	2.61–199.46

AD, autosomal dominant; AR, autosomal recessive; N/A, not available; SE, standard error.

**Table 3:** Vestibular symptoms and results of vestibular function tests for each patient with confirmed genetic variations.

	ID	Gene	Degree of Hearing Loss	Audiogram	Age of Onset	Vestibular Symptoms			Vestibular Function Test			
						Type	Duration	Recurrence	Caloric	vHIT	cVEMP	oVEMP
**AD**	YUHL195-21	*COCH*	Profound	Flat	35	Unsteadiness	Continuous	N/A	Abnormal	Abnormal	Abnormal	Abnormal
	YUHL519-21		Mild	Down-sloping	N/A	N/A	N/A	N/A	Normal	Normal	Abnormal	N/A
	YUHL5-21		Profound	Flat	N/A	N/A	N/A	N/A	Normal	N/A	N/A	N/A
	YUHL612-21		Moderate	Flat	40	Vertigo	Hours	Recurrent	Normal	N/A	N/A	N/A
	YUHL612-12		Severe	Flat	62	Unsteadiness	Continuous	N/A	N/A	N/A	N/A	N/A
	YUHL590-22		Moderate	Down-sloping	N/A	N/A	N/A	N/A	Normal	N/A	N/A	N/A
	YUHL473-21	*DIAPH1*	Moderate	Flat	55	Vertigo	Days	Recurrent	Normal	Normal	N/A	N/A
	YUHL466-21	*DMXL2*	Normal	Up-sloping	37	Vertigo	Minutes	Recurrent	N/A	N/A	N/A	N/A
	YUHL48-12	*EYA4*	Profound	Flat	N/A	N/A	N/A	N/A	Abnormal	Abnormal	Abnormal	N/A
	YUHL400-21	*GRHL2*	Moderate	Flat	50	Unsteadiness	Seconds	Recurrent	N/A	N/A	N/A	N/A
	YUHL35-21	*KCNQ4*	Moderate	Down-sloping	35	Non-vertiginous dizziness	Hours	Recurrent	N/A	N/A	N/A	N/A
	YUHL35-31		Moderate	U-shaped	6	Non-vertiginous dizziness	Hours	Recurrent	N/A	N/A	N/A	N/A
	YUHL168-21		Severe	Flat	N/A	N/A	N/A	N/A	Normal	Abnormal	Abnormal	N/A
	YUHL91-21	*MYH9*	Severe	Flat	38	Vertigo	Hours	Recurrent	Abnormal	Normal	Abnormal	Abnormal
	YUHL460-21	*MYO7A*	Mild	Reverse U-shaped	46	Non-vertiginous dizziness	Days	Recurrent	N/A	N/A	N/A	N/A
	YUHL541-21		Moderate	Up-sloping	N/A	Unsteadiness	Continuous	Recurrent	Normal	Normal	Abnormal	Abnormal
	YUHL550-21		Profound	Down-sloping	24	Non-vertiginous dizziness	Minutes	Recurrent	N/A	N/A	N/A	N/A
	YUHL628-21		Severe	Down-sloping	40	Vertigo	Hours	Single	N/A	N/A	N/A	N/A
	YUHL282-21	*NLRP3*	Moderate	Flat	25	Vertigo	Hours	Recurrent	Abnormal	Normal	Normal	N/A
	YUHL481-21		Moderate	Up-sloping	N/A	N/A	N/A	N/A	N/A	N/A	N/A	N/A
	YUHL365-21	*POU4F3*	Moderate	Flat	31	Vertigo	Days	Single	N/A	N/A	N/A	N/A
	YUHL517-21	*TECTA*	Moderate	Down-sloping	28	Non-vertiginous dizziness	Minutes	Recurrent	N/A	N/A	N/A	N/A
	YUHL292-21	*WFS1*	Moderate	Flat	58	Vertigo	Hours	Recurrent	N/A	N/A	N/A	N/A
**AR**	YUHL439-21	*CDH23*	Profound	Down-sloping	67	Non-vertiginous dizziness	Days	Recurrent	Normal	Normal	Abnormal	N/A
	YUHL434-21	*GJB2*	Moderate	Down-sloping	43	Vertigo	Minutes	Recurrent	N/A	N/A	N/A	N/A
	YUHL638-21	*MPZL2*	Moderate	Flat	N/A	N/A	N/A	N/A	N/A	N/A	N/A	N/A
	YUHL339-21	*MYO15A*	Moderate	Flat	N/A	N/A	N/A	N/A	Abnormal	Normal	Abnormal	N/A
	YUHL604-21		Severe	Down-sloping	N/A	Vertigo	N/A	Recurrent	Normal	Normal	N/A	N/A
	YUHL269-21	*SLC26A4*	Profound	Flat	8	Vertigo	Days	Recurrent	Abnormal	Normal	Abnormal	Abnormal
	YUHL283-21		Severe	Down-sloping	31	Non-vertiginous dizziness	Minutes	Single	N/A	N/A	N/A	N/A
	YUHL322-21		Profound	Down-sloping	13	Vertigo	Days	Recurrent	N/A	N/A	N/A	N/A
	YUHL480-21		Profound	Down-sloping	N/A	N/A	N/A	N/A	N/A	N/A	N/A	N/A
	YUEVA63		Profound	Flat	N/A	N/A	N/A	N/A	N/A	N/A	N/A	N/A
	YUEVA64		Profound	Down-sloping	N/A	N/A	N/A	N/A	Normal	N/A	Normal	N/A
	YUEVA98		Profound	Down-sloping	N/A	N/A	N/A	Recurrent	N/A	N/A	N/A	N/A
	YUEVA68		Profound	Down-sloping	N/A	N/A	N/A	N/A	N/A	N/A	N/A	N/A
	YUEVA85		Profound	Down-sloping	26	Vertigo	Seconds	Recurrent	Abnormal	N/A	Normal	Normal
	YUEVA101		Profound	Flat	5	Vertigo	Days	Recurrent	N/A	N/A	Normal	N/A
	YUEVA118		Profound	Flat	N/A	N/A	N/A	Recurrent	Normal	Normal	Normal	N/A
	YUEVA148-21		Profound	Flat	N/A	N/A	N/A	N/A	N/A	N/A	N/A	N/A
	YUEVA110		Severe	Flat	12	N/A	N/A	N/A	N/A	N/A	N/A	N/A
	YUEVA115		Profound	Flat	N/A	N/A	N/A	N/A	Abnormal	Normal	Normal	N/A
	YUHL430-21	*WFS1*	Normal	Up-sloping	N/A	N/A	N/A	N/A	N/A	N/A	N/A	N/A
**XD**	YUHL455-22	*COL4A6*	Moderate	Flat	N/A	N/A	N/A	N/A	N/A	N/A	N/A	N/A
**XR**	YUHL143-21	*PRPS1*	Moderate	Flat	N/A	N/A	N/A	N/A	N/A	N/A	N/A	N/A

AD, autosomal dominant; AR, autosomal recessive; cVEMP, cervical vestibular-evoked myogenic potential; oVEMP, ocular vestibular-evoked myogenic potential; vHIT, video head impulse test; XD, X-linked dominant; XR, X-linked recessive; YUHL, Yonsei University Hearing Loss.

**Table 4:** Inheritance patterns and genes associated with abnormal results of vestibular function tests.

	AD Genes (n)	AR Genes (n)	Total (%)	*p*-Value
Caloric test			22	<0.001
Unilateral	*MYH9* (1), *NLRP3* (1), Unknown (2)	*MYO15A* (1), *SLC26A4* (3), Unknown (10)	18 (81.8)	
Bilateral	*COCH* (1), *EYA4* (1)	*SLC26A4* (1), Unknown (1)	4 (18.2)	
vHIT			7	
Unilateral	Unknown (2)	Unknown (2)	4 (57.1)	
Bilateral	*COCH* (1), *EYA4* (1), *KCNQ4* (1)		3 (42.9)	
cVEMP test			26	
Unilateral	*KCNQ4* (1)	*CDH23* (1), *SLC26A4* (1), Unknown (2)	5 (19.2)	
Bilateral	*COCH* (2), *EYA4* (1), MYH9 (1), *MYO7A* (1), Unknown (7)	*MYO15A* (1), Unknown (8)	21 (80.8)	
oVEMP test			16	
Unilateral	Unknown (1)	*SLC26A4* (1), Unknown (2)	4 (25.0)	
Bilateral	*COCH* (1), *MYH9* (1), *MYO7A* (1), Unknown (7)	Unknown (2)	12 (75.0)	

AD, autosomal dominant; AR, autosomal recessive; cVEMP, cervical vestibular-evoked myogenic potential; oVEMP, ocular vestibular-evoked myogenic potential; vHIT, video head impulse test.

## Data Availability

Data are contained within the article.

## Supplementary Materials

The following supporting information can be downloaded at https://www.mdpi.com/article/10.3390/jcm13072001/s1: Table S1. The list of genes in the NGS panel. Table S2. Identified mutations and individual genotypes of the patients with vestibular phenotypes in this study. Table S3. Identified genes in this study and their expression in the vestibular organs.
